# Supplementary figures and images for: Mutations in SARS-CoV-2 are on the increase against the acquired immunity
Source: PLoS One. 2022 Jul 11;17(7):e0271305. doi: 10.1371/journal.pone.0271305 (PMC9273074; doi:10.1371/journal.pone.0271305)

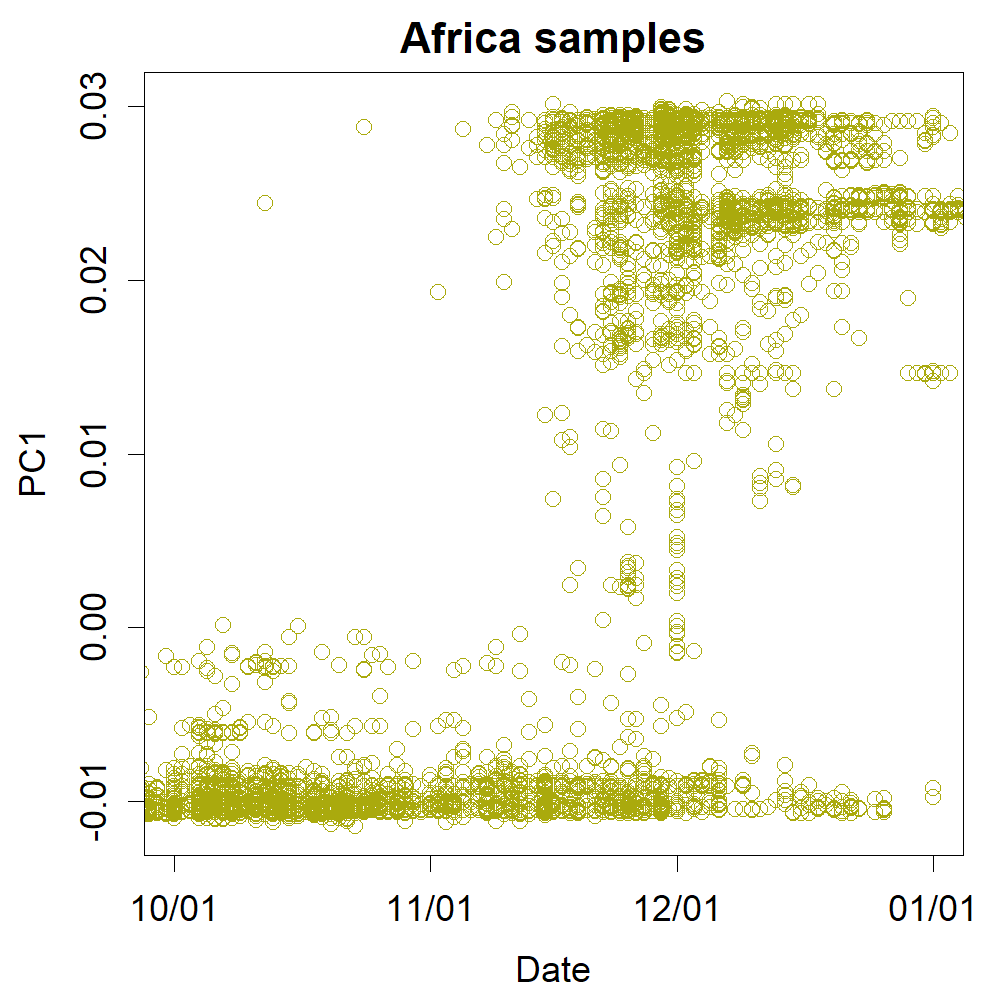

Supplement: S1 Fig — The upper third of the samples are cases with Omicron variant and the middle part is B.1.1.519. The earliest reports are from the uppermost part, which is the most mutated and infectious. The changes involved in the formation of the Omicron variant do not appear in this time series. Rather, it is more likely that a mature variant has entered the countries where these sequence testing methods are being carried out. (TIF) [file pone.0271305.s001.tif]

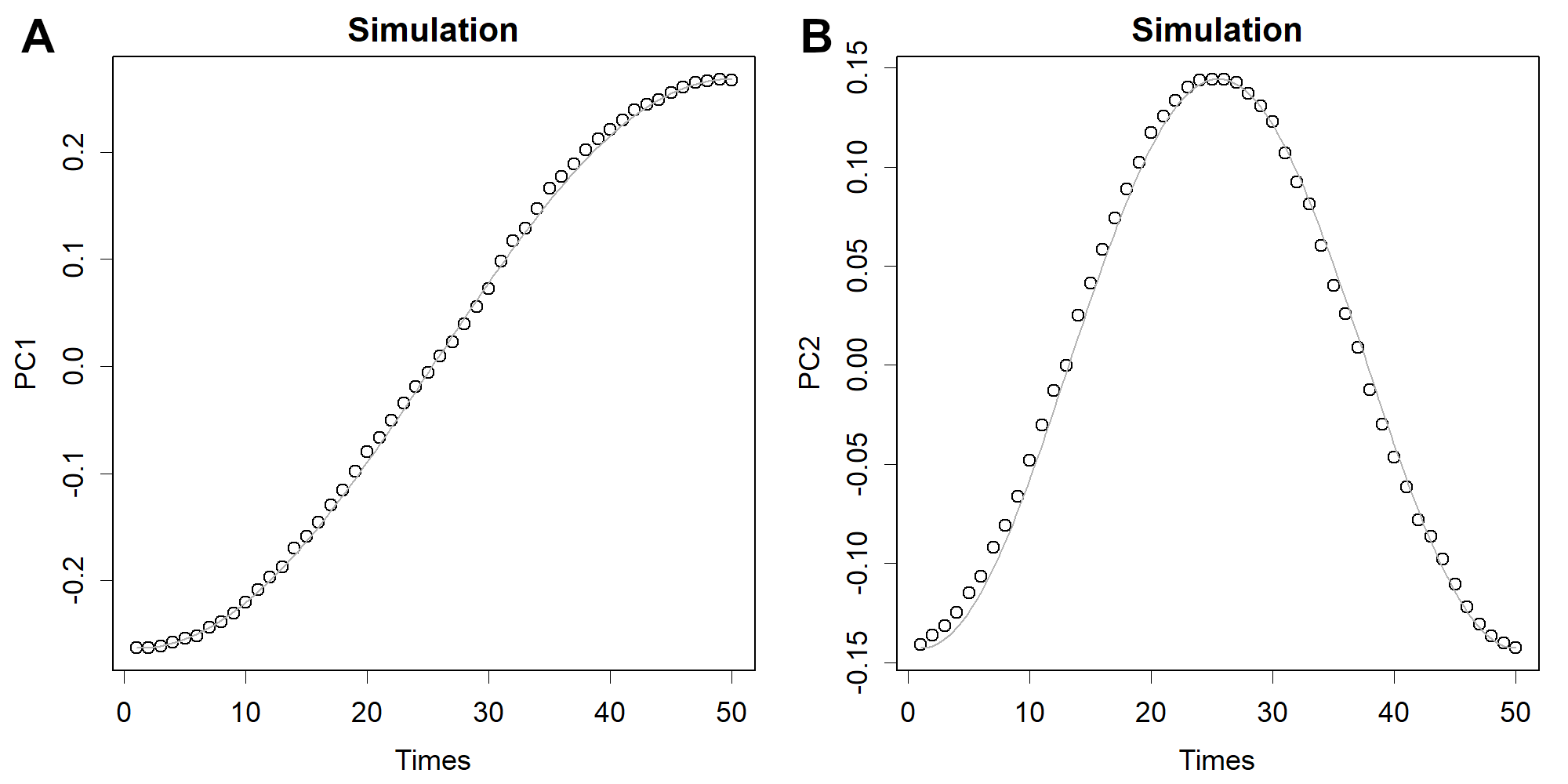

Supplement: S2 Fig — The gray line is a sine curve, PC1 is half a cycle, and PC2 is one cycle. Influenza H1N1 showed a similar pattern. The length of the base was 1e4, and the number of trials was 1000. The average of all results was used for centering before PCA. The results are scaled, but they are still much larger than those for COVID-19, which has not yet produced as many mutations. (TIF) [file pone.0271305.s002.tif]

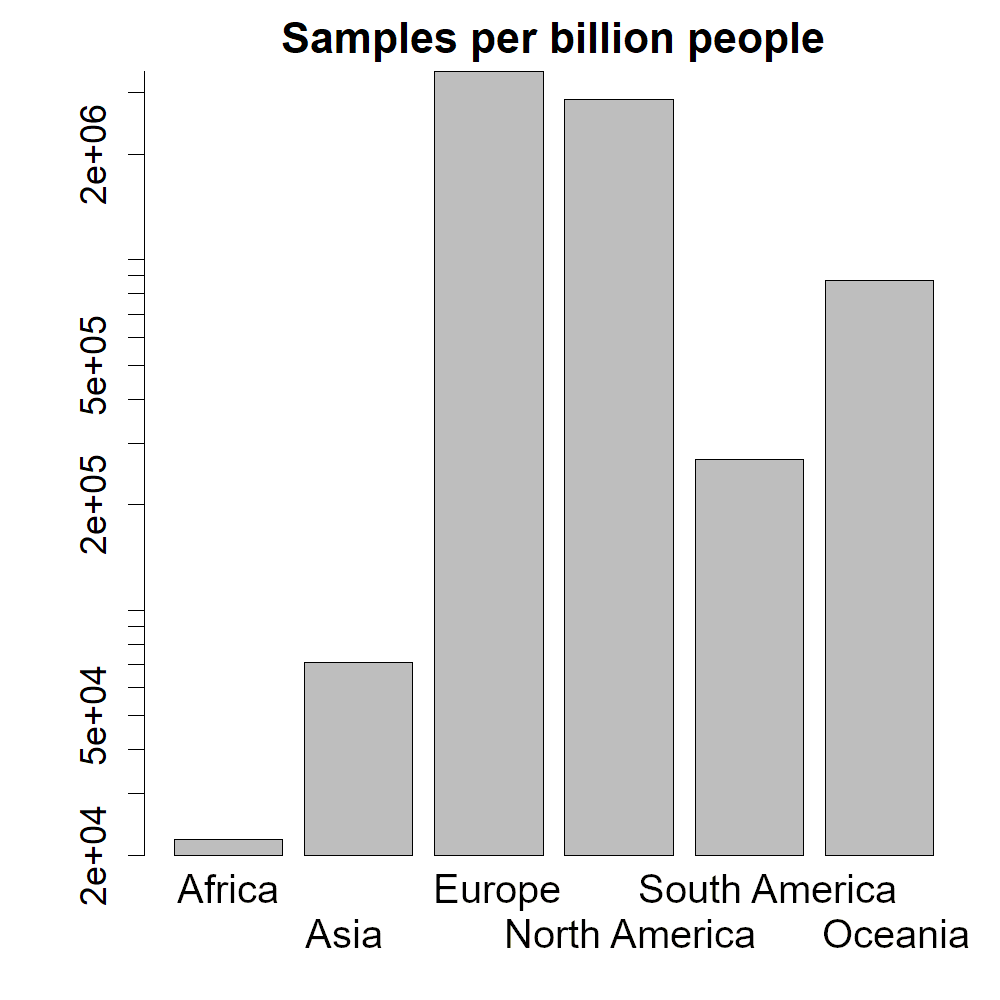

Supplement: S3 Fig — The axes are logarithmic. Africa is two orders of magnitude lower than the other regions. (TIF) [file pone.0271305.s003.tif]

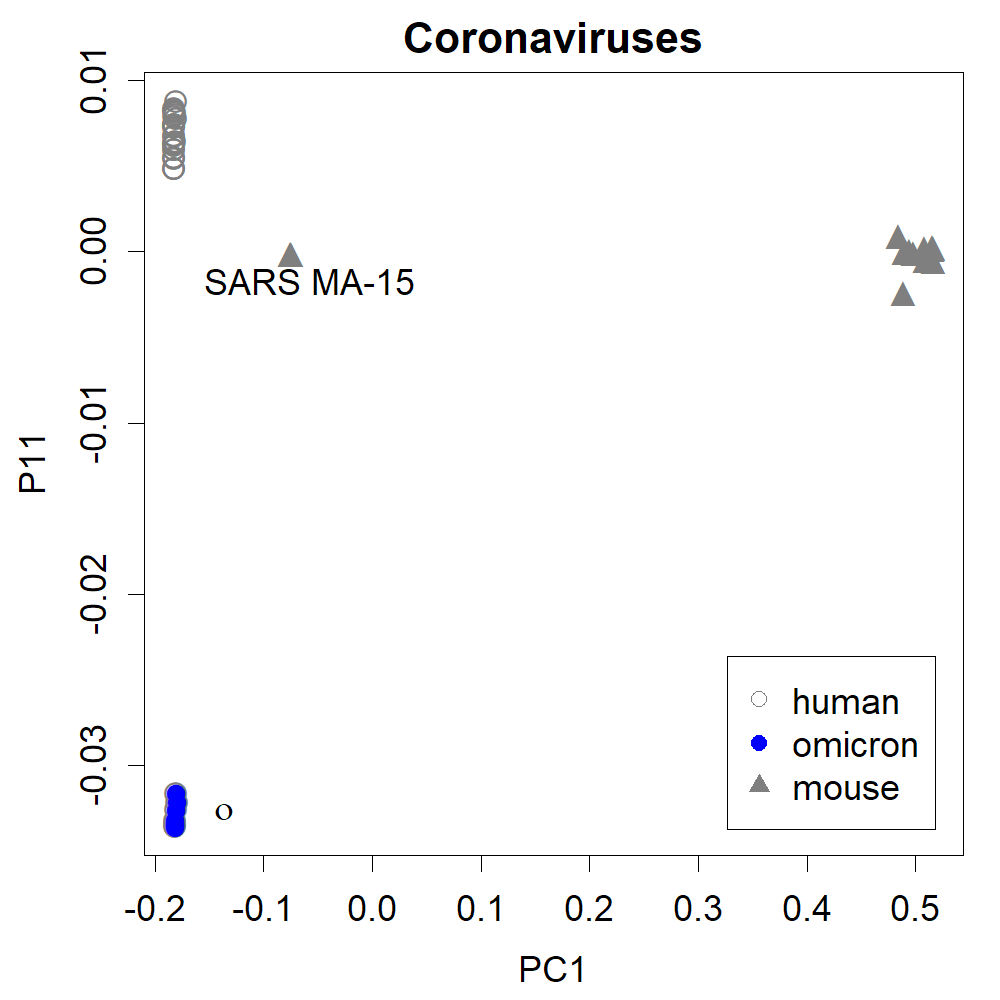

Supplement: S4 Fig — Five samples of Omicron variant were used and the axes were set from all said samples. The rodent variants were very far from the human SARS-CoV-2; a difference that appears in principal component 1 (PC1). Only one SARS variant infecting rodents appeared somewhat closer to humans. The characteristics of the Omicron variant appear in PC 11, whereas the rodent virus does not show these characteristics at all. These viruses are unrelated. (TIF) [file pone.0271305.s004.tif]

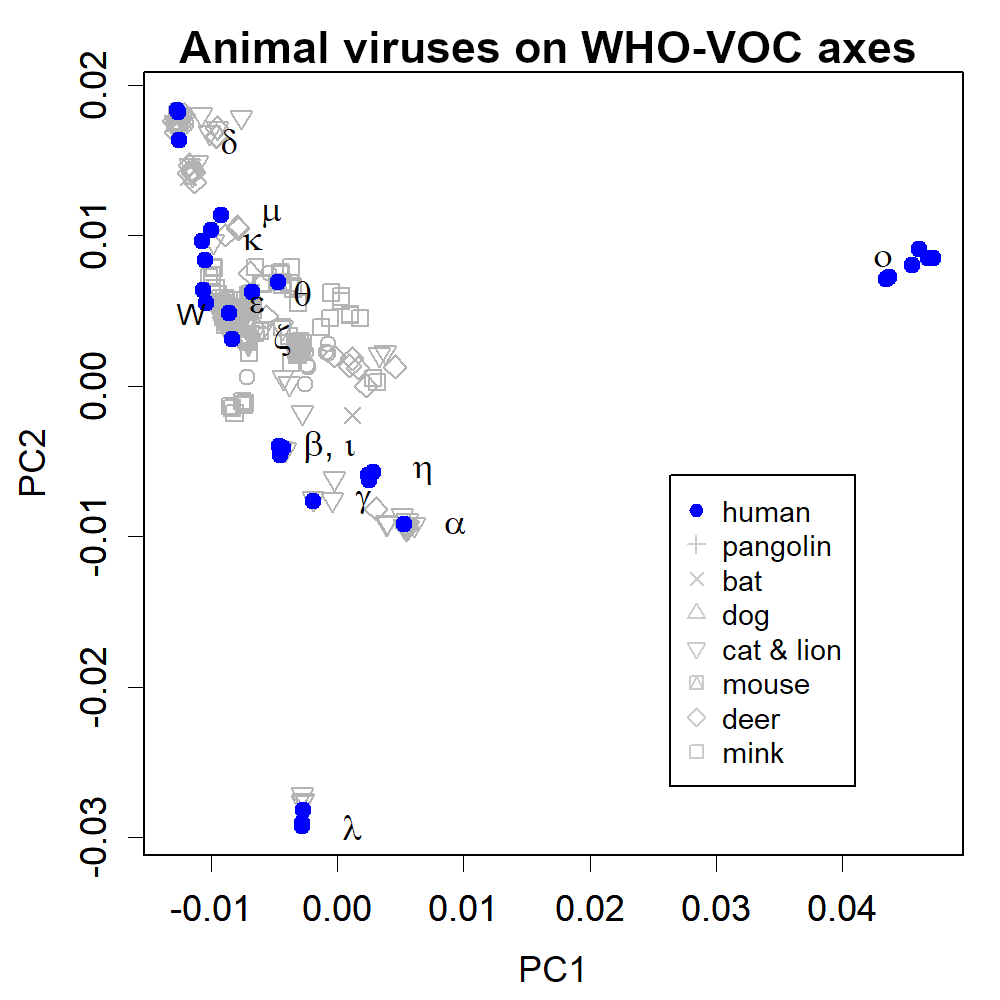

Supplement: S5 Fig — Blue is the WHO-VOC. Each variant infected animals to a degree; the most recent. (TIF) [file pone.0271305.s005.tif]
