# Supplementary material for: Mutations in SARS-CoV-2 are on the increase against the acquired immunity
Source: PLoS One. 2022 Jul 11;17(7):e0271305. doi: 10.1371/journal.pone.0271305 (PMC9273074; doi:10.1371/journal.pone.0271305)
Supplement: Attachment — Submitted filename: Manuscript PONE-D-22-05674 Review Report from Rashed Noor.pdf [file pone.0271305.s007.pdf]

Manuscript #: PONE-D-22-05674
Title: Increasing SARS-CoV-2 mutations against vaccination-acquired immunity
Authors: Tomokazu Konishi
Article type: Research Article

The manuscript written by Dr. Tomokazu Konishi focused a major issue about COVID-19 booster vaccination specially to eradicate the effects of Omicron variants. The necessity of vaccination, about the commencement of herd immunity to prevent the mass public health from a pandemic are clearly stated in this manuscript. The objectives, results, and interpretations have been clearly given. Methods are sound as well. The knowledge gained from this work is of global interest. However, the manuscript needs language improvement. In some places, the superficial terms/ phrases have been used. If the manuscript is language edited and improved technically, it may warrant publication because of its merit and sound presentation quality. Therefore, the author is requested to fix some points as appended below.

In the Abstract section, the author may change the term epidemic to pandemic. All the VOCs should be mentioned. Also, the term “weaker variant” may be explained in a sentence in course of amino acid replacements/ deletions.

The Key Word section may add “booster dose”.

As a single author, in the Competing Interest section, “authors” cannot be used.

Introduction
Line 31: COVID-19 pandemic; this is not epidemic. Indeed, the first sentence in lines 31-32 is not clear and attractive. Please revise it.

Line 32: What does the author mean by the word “postulate”? Please use appropriate English.

Lines 37-38: Mention the potent variants with lineages with timeline in short. Give reference.

Line 39: How about the variants of interest (VOI)?

Line 46: That warning is a past tense indeed. Author must clearly discuss why the vaccines were in question in terms of their efficacy against the variants, especially against the Delta and Omicron variants. Then it was resolved by the application of the third dose; i.e., the so called booster dose. The memory B cells were activated by this booster. Author should put such explanation here with appropriate references.

Line 50: Omicron variant has two subtypes. Author should mention the names specifically with the number and position of mutations individually.

Line 53: Why “we”? Author himself is the author. Also, the manuscript should be written in passive form.

Lines 58-60: Author should take care of the “tense”. The language should be fixed with a native speaker as I have noticed so far after reading the Introduction section.

Materials and Methods

Line 63: Be more specific about the nucleotide sequence.

Line 77: What is “weighting errors”? Check the grammar.

Line 100: Fig. 2A shows NOT reflects

Lines 106-107: Give specific example of this sentence.

Line 114: What is meant by “each epidemic”?

Line 116: Author should also include the viral assortment in the H1N1 influenza.

Lines 121-122: I didn’t understand this sentence. Please write more specifically with immunological terms.

Lines 127-128: If the information is lacking, I think author should try to find out it. If he can’t, it’s better to delete this sentence. To my knowledge, no information is lacking regarding COVID-19 pandemic. Author should be more careful to include all the relevant data.

Line 149: Fix the typo error.

Lines 153-154: write as pandemic, and NOT epidemic.

Line 159: A simple sequence of mutations can be written here in case of the initial variant. Readers may understand well.

Line 163: Please address the mutations in Omicron variant quantitatively.

Lines 169-170: Re-write this sentence a simpler form. The word “farthest” is not a suitable one.

Line 175: Lambda and Delta ---- newer variants? Isn’t it contradictory? Please give explanation.

Discussion

Line 186: “discontinuous” in what sense? Author tried to explain that but this word is not suitable.

Line 200: Please include the frequency.

Line 202: “The USA and the UK are the most prolific sequencers.” --- This sentence is actually incomplete.

Line 209: How the author defines the monovalent vaccines? Author can try with the chemical composition of specific vaccines under trial.

Line 226: mRNA vaccines

Lines 231-233: Can author put some lines to explain the identical mutations in the Omicron variant?

Conclusion

Line 250-251: Weak sentence. I can’t get it actually.

Acknowledgement:

Line 257: Why “we”?
