# Supplementary material for: Mutations in SARS-CoV-2 are on the increase against the acquired immunity
Source: PLoS One. 2022 Jul 11;17(7):e0271305. doi: 10.1371/journal.pone.0271305 (PMC9273074; doi:10.1371/journal.pone.0271305)
Supplement: Attachment — Submitted filename: Review comments ST.docx [file pone.0271305.s006.docx]

**Increasing SARS-CoV-2 mutations against vaccination-acquired immunity**

- Tomokazu Konishi

Review comments:

| **Line number** | **Comment** |
| --- | --- |
| **Line 16-17** | “Monovalent vaccines using RNA or adenoviruses have successfully controlled the COVID-19 epidemic in many countries”  The above statement may be refined as “Monovalent vaccines using RNA or adenoviruses have provided substantial protection against the COVID-19 epidemic in many countries” |
| Line 18-19 | The statement says the Omicron, “in particular”, put pressure on the healthcare system. But, weren’t hospitalizations comparatively lower for Omicron waves? |
| Line 26 | Will “causing another pandemic wave” be more appropriate? |
| Line 79 | Typo: Omicron variants |
| Line 162 | Typo: …which are the surface proteins…  Is N considered a surface protein? |
| Line 215 | Maybe the potential dangers can be specified |
| Line 224 | But all viral proteins are not antigenic! |
| Line 217-228 | How will the effectiveness of animal-adapted viral vaccines be different from currently available inactivated vaccines?  Is there any data to show the effectiveness/challenges/limitations of whole inactivated virus vs monovalent vaccines? |
| Line 246-253 | Lines 229-236 is repeated in the Conclusion section |
