# Supplementary material for: Mutations in SARS-CoV-2 are on the increase against the acquired immunity
Source: PLoS One. 2022 Jul 11;17(7):e0271305. doi: 10.1371/journal.pone.0271305 (PMC9273074; doi:10.1371/journal.pone.0271305)
Supplement: Attachment — Submitted filename: ResponsetoReviewers.docx [file pone.0271305.s008.docx]

# # Reviewer 1

Manuscript #: PONE-D-22-05674

Title: Increasing SARS-CoV-2 mutations against vaccination-acquired immunity

Authors: Tomokazu Konishi

Article type: Research Article

The manuscript written by Dr. Tomokazu Konishi focused a major issue about COVID-19 booster vaccination specially to eradicate the effects of Omicron variants. The necessity of vaccination, about the commencement of herd immunity to prevent the mass public health from a pandemic are clearly stated in this manuscript. The objectives, results, and interpretations have been clearly given. Methods are sound as well. The knowledge gained from this work is of global interest. However, the manuscript needs language improvement. In some places, the superficial terms/ phrases have been used. If the manuscript is language edited and improved technically, it may warrant publication because of its merit and sound presentation quality. Therefore, the author is requested to fix some points as appended below.

In the Abstract section, the author may change the term epidemic to pandemic. All the VOCs should be mentioned. Also, the term “weaker variant” may be explained in a sentence in course of amino acid replacements/ deletions.

I appreciate your observation and comment. I have revised some of the sentences accordingly. However, I would like to avoid mentioning the other numerous VOC, since the Omicron variant is the focus of this article. This is also to satisfy the request of not using abbreviations (VOC) in the abstract.

The Key Word section may add “booster dose”.

I added this word.

As a single author, in the Competing Interest section, “authors” cannot be used.

I replaced the sentence.

Introduction

Line 31: COVID-19 pandemic; this is not epidemic. Indeed, the first sentence in lines 31-32 is not clear and attractive. Please revise it.

I revised the sentence.

Line 32: What does the author mean by the word “postulate”? Please use appropriate English.

I apologize for the wrong word choice. I have revised ‘epidemic’ to ‘pandemic’ and improved the language for easy understanding and better readability throughout the manuscript.

Lines 37-38: Mention the potent variants with lineages with timeline in short. Give reference.

I have included the potent variants and the references as requested.

Line 39: How about the variants of interest (VOI)?

I have included this.

Line 46: That warning is a past tense indeed. Author must clearly discuss why the vaccines were in question.

The present perfect tense would be more correct since the question is in the present, and I have corrected it. I also have added a figure to explain why the effectiveness of vaccines has been questioned, presenting the data in a new Fig. 1.

Line 50: Omicron variant has two subtypes. Author should mention the names specifically with the number and position of mutations individually.

This is quite technical and complex because the PANGO system and that used in this work have no relationship. Currently, the WHO has divided Omicrons into about five types. In Fig. 2, it is clearly shown that the biggest gap is the presence or absence of an insert, but this difference is not related to the types in the PANGO system. I have explained this situation in L140 and my thought about it in L279; I suppose it is clear that PCA is the superior method.

I have published several articles on the evolution of coronaviruses; however, I avoided to mention their classifications or nomenclature. This is because I believe that the classifications are not objective and thus difficult to be explained by science. We cannot determine the boundary between the variants or species with the objectivity required for science. Rather, the classifications would be more of an act of human convenience. The classifications should be defined to facilitate the work of those in the field. In this case, it is more important from a disease control point of view, i.e., considering the country affected by the pandemic etc.

Line 53: Why “we”? Author himself is the author. Also, the manuscript should be written in passive form.

I have replaced them. Also, I have reduced the frequency of using active voice (for example, L25, L30, L68, L98, L106, L116, and L117).

Lines 58-60: Author should take care of the “tense”. The language should be fixed with a native speaker as I have noticed so far after reading the Introduction section.

This article has been edited by a native and professional English editor. I will ask the company to review it again. I understand that we use the past tense for the ‘results of experiments conducted in the past’, the present tense for ‘statements of fact’, and the future tense for ‘probable predictions that may happen’. I apologize if this was not implemented appropriately.

Materials and Methods

Line 63: Be more specific about the nucleotide sequence.

It has been specified.

Line 77: What is “weighting errors”? Check the grammar.

I have added an explanation while avoiding this expression.

Line 100: Fig. 2A shows NOT reflects

I have changed the wording.

Lines 106-107: Give specific example of this sentence.

I have added an explanation using examples.

Line 114: What is meant by “each epidemic”?

I have changed the wording.

Line 116: Author should also include the viral assortment in the H1N1 influenza.

I have added the assortment.

Lines 121-122: I didn’t understand this sentence. Please write more specifically with immunological terms.

I have added a more detailed explanation. Moreover, I have quoted from a previous article on this subject.

Lines 127-128: If the information is lacking, I think author should try to find out it. If he can’t, it’s better to delete this sentence. To my knowledge, no information is lacking regarding COVID-19 pandemic. Author　should be more careful to include all the relevant data.

I used all the information registered on GISAID. I have added this sentence to make it clear that it is inserted as a precursor to the ‘sequence that has been reported with bias’, which I will describe later.

Line 149: Fix the typo error.

I appreciate pointing out this.

Lines 153-154: write as pandemic, and NOT epidemic.

I have replaced those.

Line 159: A simple sequence of mutations can be written here in case of the initial variant. Readers may understand well.

I have given one clear example of mutation. I hope it will help you understand the principle of PCA.

Line 163: Please address the mutations in Omicron variant quantitatively.

I have added a citation to the figure. Unfortunately, *addressing Omicron variant quantitatively* is technically impossible. A more detailed information than the broadly summarized Fig. 4 can be obtained by looking at the sequence and to some extent, from the PC_gene. Such a situation was also explained in L217-220.

Lines 169-170: Re-write this sentence a simpler form. The word “farthest” is not a suitable one.

I have rewritten that part (L221).

Line 175: Lambda and Delta ---- newer variants? Isn’t it contradictory? Please give explanation.

I have added the citation to figures (L229).

Discussion

Line 186: “discontinuous” in what sense? Author tried to explain that but this word is not suitable.

I have rewritten that part.

Line 200: Please include the frequency.

I have added the information (L268).

Line 202: “The USA and the UK are the most prolific sequencers.” --- This sentence is actually incomplete.

I have rewritten that part. (L272)

Line 209: How the author defines the monovalent vaccines? Author can try with the chemical composition of specific vaccines under trial.

Novavax and Shionogi vaccines use proteins, the AstraZeneca vaccine uses adenovirus vectors, and Pfizer and Moderna vaccines use mRNA. These probably differ in effectiveness and especially in the magnitude of adverse reactions. I am interested in this, but I do not have enough data or articles to cite and describe in detail. Therefore, I simply wrote "monovalent" here. I hope protein vaccines will have fewer adverse reactions, but this is only a desire at present.

Line 226: mRNA vaccines

I have rewritten that part (L63, 249).

Lines 231-233: Can author put some lines to explain the identical mutations in the Omicron variant?

I have rewritten it with specific examples.

Conclusion

Line 250-251: Weak sentence. I can’t get it actually.

I have rewritten that part.

Acknowledgement:

Line 257: Why “we”?

I have rewritten that part.

# # Reviewer 2

Review comments:

| **Line number** | **Comment** |
| --- | --- |
| **Line 16-17** | “Monovalent vaccines using RNA or adenoviruses have successfully controlled the COVID-19 epidemic in many countries”  The above statement may be refined as “Monovalent vaccines using RNA or adenoviruses have provided substantial protection against the COVID-19 epidemic in many countries”  I appreciate this comment, and I have replaced the sentence. |
| Line 18-19 | The statement says the Omicron, “in particular”, put pressure on the healthcare system. But, weren’t hospitalizations comparatively lower for Omicron waves?  I have added to this section that some countries have changed their policies because the infectiousness of Omicron was very severe.  Unfortunately, the actual burden on medical care remains high. To make this situation clear, I have added a brief summary of the actual situation in Japan as a new Fig. 1 as well as a brief discussion. Unfortunately, the actual burden of medical care remains high. |
| Line 26 | Will “causing another pandemic wave” be more appropriate?  I have changed the expression to the present perfect tense. |
| Line 79 | Typo: Omicron variants  I appreciate this point out. I have fixed it. |
| Line 162 | Typo: …which are the surface proteins…  Is N considered a surface protein?  I appreciate your observation, and I have corrected it. N is on the surface of the nucleocapsid, and not the virus. |
| Line 215 | Maybe the potential dangers can be specified  I appreciate pointing this out. I have added a note. |
| Line 224 | But all viral proteins are not antigenic!  I have rephrased the expression because no one knows this presently. It may be known in the future once the data on the severity of the disease among those that have been infected become available. However, if we can deduce this from the case of influenza, we can expect it to be antigenic, as is written here.  On a very personal note, I have been infected once with the Alpha variant. Last week, my daughter was infected with the Omicron variant, and I stayed home to take care of her. I probably got infected again as I felt very tired but did not develop the disease. I am avoiding the booster shots; therefore, this might be due to the developed immunity from the previous infection. |
| Line 217-228 | How will the effectiveness of animal-adapted viral vaccines be different from currently available inactivated vaccines?  Is there any data to show the effectiveness/challenges/limitations of whole inactivated virus vs monovalent vaccines?  I appreciate this comment. I have added some general information about the types of vaccines. Although the merits and demerits of monovalent vaccines, especially the mRNA vaccines, will become apparent in the future, I have cited some additional findings on the adverse reactions that have been frequently reported recently. |
| Line 246-253 | Lines 229-236 is repeated in the Conclusion section  This was my mistake. My apologies. I have replaced the sentence. |
